# A Service Evaluation of the Experiences of Patients With Functional Neurological Disorders Within the NHS

**DOI:** 10.3389/fneur.2021.656466

**Published:** 2021-05-31

**Authors:** Shauna O'Keeffe, Ibrahim Chowdhury, Anila Sinanaj, Iberedem Ewang, Camilla Blain, Tiago Teodoro, Mark Edwards, Mahinda Yogarajah

**Affiliations:** ^1^Atkinson Morley Regional Neuroscience Centre, St. George's University Hospitals National Health Service Foundation Trust, London, United Kingdom; ^2^Neuroscience Research Centre, Institute of Molecular and Clinical Sciences, St. George's University of London, Cranmer Terrace, London, United Kingdom; ^3^National Hospital for Neurology and Neurosurgery, London, United Kingdom; ^4^Department of Experimental and Clinical Epilepsy, University College London Institute of Neurology, London, United Kingdom

**Keywords:** functional neurological disorder, conversion disorder, patient-reported experience measure, long-term neurological conditions, patient experience, service evaluation, standards of care, NHS

## Abstract

Previous research into Functional Neurological Disorder (FND) has shown that there are significant barriers in providing patient-centred care. However, there has been no specific research into whether patient experiences of care for FND meet the current standards of care. This study aimed to investigate the types of problems experienced by FND patients, and whether these differed to patients with multiple sclerosis (MS). FND (*n* = 40) and MS patients (*n* = 37) were recruited from NHS tertiary neurology clinics and completed questionnaires on their experiences of health care services. Significant differences in experiences of care between the two patient groups were found, with FND patients reporting significantly more problems in their diagnosis and treatment (*p* = *0.003)*, patient-centred care *(p* < *0.001)*, relationships with healthcare professionals *(p* < *0.001)*, and in accessing community care *(p* = *0.001)*. Limitations include a small sample size, specificity to a single centre, and cross-sectional design. The results suggest that current care for FND patients is not meeting expected standards for long-term neurological conditions, highlighting the need for structured care pathways and patient-centred care.

## Introduction

Long-term neurological conditions (LTNCs) are conditions arising from damage to or a disease of the nervous system that confers a life-long impact to the individual (1). They are becoming increasingly prevalent, leading to an increasing utilisation of health and social care services to diagnose and manage these conditions (2).

Many patients with LTNCs, however, identify unmet needs and problems with care provision. A large-scale survey of patients with LTNCs in the UK found multiple issues, including delays in diagnosis and referral, a lack of structured care plans, and problems in accessing services or treatments (3). These unmet needs may contribute to increased care burden and costs for patients as they seek the use informal and community-based care instead (4–6).

Functional neurological disorder (FND; also referred to as conversion/psychogenic disorder) refers to the presence of neurological symptom (e.g., seizure-like events, limb weakness, etc.) that are involuntary, but are incongruent with symptoms cause by neurological disease. They very commonly have a chronic course, thus constituting a LTNC for many patients. Symptoms of FND are the second most common reason for referral to neurology clinics (7) and patients with FND experience high levels of disability and emotional distress which are equal to, or even greater than, other LTNCs (8, 9). The prognosis of FND in general with current management is often poor (10), although significant rates of improvement have been found depending on the specific groups of functional symptoms. Additionally, he quality of life of people with FND has been found to be lower compared to other LTNCs such as Parkinson's disease (11). This suggests there are issues present in the way in which FND is managed and how FND patients are supported in current health and social care systems.

People with FND often have to be seen multiple times before a definite diagnosis is made, causing marked delays in diagnosis delivery and uncertainty around the diagnosis (12, 13). The management of FND requires specialist multidisciplinary care across many services, including both health and social care services within community and hospital settings, and spanning both physical and mental health providers (14, 15). This can be challenging due to differing approaches to management from different healthcare professionals and between services, the lack of resources available for FND care, and there being a lack of knowledge about FND within healthcare services (16, 17). Further, professionals in primary care settings, such as GPs, are often the facilitators via which specialist management can be accessed, and thus the attitudes and ethical presumptions of these primary care professionals can provide additional barriers in accessing appropriate care. Negative perceptions of patients with FND amongst a variety of healthcare professionals (18–20) arising from a lack of knowledge about the condition and suspicion of feigning may also impact the extent to which patient-centred care is provided and lead to greater stigmatisation of these patients (21). These barriers may therefore impact on the experience of people with FND as they navigate health systems in order to obtain the help they need.

However, whilst the need to improve the care pathway and patient experience for FND has been previously identified (14, 22, 23), there has been limited research into how FND patients experience the care they receive. Whilst there has recently been key developments in identifying the need for more patient-reported outcome measurements (PROMs) for people with FND and in increasing the validity of these measurements (24, 25), there has been little development in patient-reported experience measures (PREMs). Thus, the subjective experiences of care that people with FND experience, and whether this experience meets the standards of care for LTNCs as outlined in clinical guidelines and national policies such as the National Service Framework, has not previously been explored.

The National Service Framework for Long Term Conditions (NSF) is a long-term 10 year strategy developed in the UK in order to improve the quality of care provided for patients with LTNCs and provide coordinated, multidisciplinary, and patient-centred care to manage these conditions (1). It sets out quality requirements relating to the diagnosis, management, and support for patients with LTNCs within the UK National Healthcare Service (NHS).

In order to measure LTNC patients' experiences of the care they receive, Peters et al. (26) developed the patient self-report Health and Social Care Questionnaire based on the NSF quality standards and relevant National Institute for Health and Care Excellence (NICE) guidelines for managing LTNCs. Their study found significant issues in the implementation of the NSF quality requirements in the care of three common LTNCs (multiple sclerosis (MS), motor neurone disease, and Parkinson's disease). Significant differences in the types and frequency of problems in care between the three LTNCs were also found.

To date, there have been no studies conducted to assess the quality of the health and social care that FND patients receive as compared to other LTNCs. By comparing FND patients with MS patients, who are similar in demographic characteristics (including a female preponderance, younger age of onset than other LTNCs, and similarly high levels of disability) (7), issues that FND patients specifically experience within the NHS can be elucidated. These insights can be used to inform changes in clinical practise and care pathways.

This study therefore aimed to compare the experience of FND patients with MS patients within the NHS using the patient-reported Health and Social Care questionnaire developed by Peters et al. (26) in order to explore whether there were differences in the frequency and types of problems experienced between the two patient groups. It was predicted that FND patients would report a higher frequency of problems in their experience of care services than MS patients in all domains, and that these differences would be especially marked in areas pertaining to patients' relationships with health and social care professionals.

## Materials and Methods

### Ethics

Ethical review and approval was not required for the study on human participants in accordance with the local legislation and institutional requirements. Written informed consent from the patients was not required to participate in this study in accordance with the national legislation and the institutional requirements.

### Participants

Seventy seven participants (40 people with FND and 37 with MS) were prospectively and sequentially recruited from a tertiary neurology service at an NHS hospital over the course of 3 months. Patients recruited were new attendees at these tertiary clinics, to which patients are typically referred to for refinement in management planning, having previously received a diagnosis and initial management elsewhere.

At their attendance to these FND and MS clinics, they were provided with questionnaires and asked to complete them whilst waiting for the consultation. Patients were explicitly told they had the right to decline completion of the questionnaire. All data was anonymously collected and stored in compliance with the principles of the Data Protection Act (1998). This project was registered and approved as an audit/service evaluation with St George's University Hospital audit department.

### Materials

Two questionnaires were used in this audit: the Experiences of Health and Social Care Questionnaire and the 5-level EQ-5D questionnaire (EQ-5D-5L). The Experience of Health and Social Care Questionnaire was developed by Peters et al. (26) to measure patients' experiences of care for their LTNC as compared to the quality standards set out by the NSF requirements and relevant NICE guidelines.

The EQ-5D-5L is a standardised tool that measures health-related quality of life using 5 dimensions: mobility, self-care, usual activities, pain/discomfort, and anxiety/depression (27). It also asks patients to provide a general health score on a scale (0 being the worst health imaginable and 100 the best) and is used routinely within clinical practice. The EQ-5D-5L questionnaire was used to assess self-rated quality of life in people with MS and FND, as well as to highlight potential confounding factors.

### Data Coding and Analysis

#### Data Coding

Responses on the Experiences of Health and Social Care Questionnaire were recoded into binary “problem” scores, with 0 generally indicating the absence of a problem/needs being partially or completely met, and 1 generally indicating the presence of a problem/needs not being met. Responses of “I am not sure” or “Not applicable” were omitted from analysis.

Items on the EQ-5D-5L questionnaire were coded in an ordinal manner, with 0 indicating no problem, 1 indicating a slight difficulty, 2 indicating a moderate difficulty, 3 indicating a severe difficulty, and 4 indicating an extreme difficulty or inability.

Summary problem scores for each domain were calculated by summing the total of “problem” items within each domain, which were then used to calculate a total problem score across all domains.

#### Statistical Analysis

The recoded data was analysed using the statistical program SPSS (version 26.0; IBM Corp, 2019). Where data was normally distributed and assumptions of statistical tests were met, independent samples *t*-tests and Pearson's chi-square tests were used to make between-groups comparisons between MS and FND patient data. Where data violated the assumption of parametric tests, non-parametric tests in the form of Mann-Whitney and Fisher exact tests were used instead. A false discovery rate correction was applied to account for multiple-testing issues, resulting in a Benjamini-Hochberg adjusted *p*-value which was used to determine if the original *p*-values remained significant (28). The significance level for all main statistical analyses was set at α = 0.05.

## Results

### Demographic Characteristics

The median age (FND = 45; MS = 47) and the gender proportions for each patient group (FND = 60% female and 37.5% male; MS = 62.2% female and 37.8% male) were not significantly different.

### Summary Problem Scores

**Figure 1:**
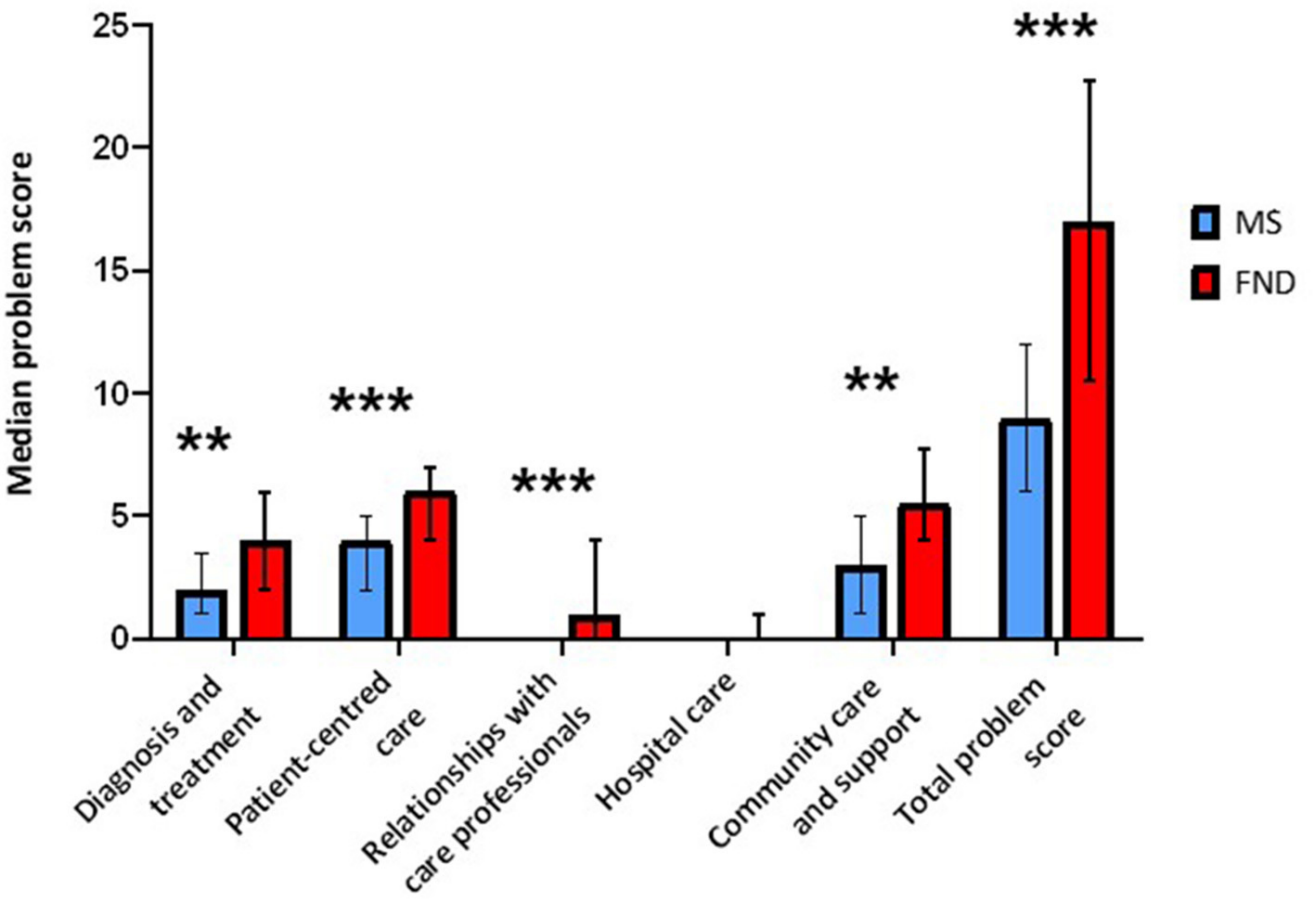
Median problem scores for each patient group across all domains and in total. There were significant differences between the patient groups for all domains except the hospital care domain. Error bars represent the upper and lower quartiles of the median asterisks denote a significant difference in medians between the patient groups at the *p* < 0.050 level, with ** indicating significance at the *p* < 0.010 level and *** indicating significance at the *p* < 0.001 level.

Respondents with FND had significantly higher total median problem scores (Mdn = 17.0) compared to respondents with MS (Mdn = 9.0, *p* < *0.001)*. The median problem scores for FND were significantly higher than for MS across the majority of the domains (Figure 1), including diagnosis and treatment (Mdn = 4.0 for FND and Mdn = 2.0 for MS; *U* = 1027.0, *p* = 0.003), patient-centred care (Mdn = 6.0 for FND and Mdn = 4.0 for MS; *U* = 1145.5, *p* <0.001), relationships with care professionals (Mdn = 1.0 for FND and Mdn = 0.0 for MS; *U* = 1111.5, *p* < 0.001), and community care and support (Mdn = 5.5 for FND and Mdn = 3.0 for MS; *U* = 1060.5, *p* = 0.001). The difference in median problem scores was not significant for the hospital care domain, although this is likely due to the limited number of patients who had been admitted to hospital in the sample (*n* = 34).

### Diagnosis and Treatment

The results of the diagnosis and treatment domain are summarised in Table 1. Respondents with FND reported waiting significantly longer than respondents with MS for both a specialist consultation (*p* < 0.001) and a definite diagnosis (*p* = 0.001). They also reported significantly more than MS respondents that they lacked information about side effects of their prescribed medication(s) (*p* = *0.010)*. There were no significant differences in regards to how their diagnosis was communicated, whether a follow-up appointment was offered, the level of information given about their condition, the use and review of medication, and whether self-management strategies were given. There were no significant differences in problems reported in receiving nutritional and respiratory support, yet it should be noted that over half of FND patients (59.5%) and 44.1% of MS patients reported needing but not being offered nutritional support, suggesting there was a large proportion of both FND and MS patients whose needs were not being met in this regard.

**Table 1:** Patients reporting a problem regarding the diagnosis and treatment of their neurological condition.

	**Patients reporting needs not met**			
	**Total**	**MS**	**FND**	
	***N (%)***	***n (%)***	***n (%)***	***p***
**Diagnosis**				
Time from GP consultation to specialist consultation (> 6 months)	36 (52.2)	7 (22.6)	29 (76.3)	<0.001^***^
Time from initial GP consultation to definite diagnosis (> 12 months)	38 (62.3)	12 (41.4)	26 (81.3)	0.001^**^
Diagnosis communicated sympathetically and empathetically	11 (21.2)	3 (10.3)	8 (34.8)	**0.036**
Follow-up appointment offered	15 (27.8)	6 (19.4)	9 (39.1)	0.109
All information about neurological condition given	23 (51.1)	10 (41.7)	13 (61.9)	0.175
**Medication (only patients taking medication) & Treatment**				
Adequate review of medication	11 (35.5)	4 (28.6)	7 (41.2)	0.364
Sufficient information about how to take medication	2 (6.9)	0 (0.0)	2 (13.3)	0.259
Sufficient information on medication side effects	14 (46.7)	3 (21.4)	11 (68.8)	0.010^*^
Given support to develop self-management strategies	47 (67.1)	26 (78.8)	21 (56.8)	0.050
Offered support with nutrition where needed	37 (52.1)	15 (44.1)	22 (59.5)	0.196
Offered respiratory support where needed	18 (25.4)	6 (17.6)	12 (32.4)	0.153

*Bolded values indicate statistical significance at the level of p < 0.05*.

* Indicate values that remained significant after a false discovery rate correction, with ^*^Indicating significance at the p < 0.050.

** *Indicating significance at the p < 0.010 level*.

*** *Indicating significance at the p < 0.001 level*.

*MS, Multiple Sclerosis; FND, Functional Neurological Disorder*.

*Values written in parenthesis are percentages*.

### Patient-Centred Care

The results of the patient-centred care domain are summarised in Table 2. FND respondents reported more difficulty in accessing health care professionals *(p* < *0.010)* and specialist clinics (*p* < 0.010). In regards to planning their care, significantly more FND than MS respondents felt that there was a lack of coordination between health and social care services (*p* < 0.010), that they were not involved in their care decisions *(p* < *0.001)*, and that their wishes were not taken into account *(p* < *0.001)*. It is notable that no respondents with MS reported the latter problem, as compared to 60% of respondents with FND. There were similar proportions of respondents in both groups who reported problems in regards to not seeing any healthcare professional within the past 12 months, not having a named healthcare professional or care coordinator to contact, not having a formal care plan (including problems in this not being adequately updated), and in not receiving information about their neurological condition.

**Table 2:** Patients reporting a problem regarding the health and social care of their neurological condition.

	**Patients reporting needs not met**			
	**Total**	**MS**	**FND**	
	***N (%)***	***n (%)***	***n (%)***	***p***
Seen any healthcare professional within the last 12 months	6 (7.8)	3 (8.1)	3 (7.5)	0.624
Experienced difficulty in seeing healthcare professionals in the last 12 months	48 (70.6)	17 (53.1)	31 (86.1)	0.003^**^
Attended a specialist clinic with the last 12 months	37 (52.9)	11 (33.3)	26 (70.3)	0.002^**^
Assigned a named healthcare professional to contact when needs change	50 (79.4)	23 (71.9)	27 (87.1)	0.136
Assigned a single health or social care professional who coordinates care	50 (87.7)	23 (85.2)	27 (90.0)	0.439
Good coordination between health and social care services in planning care	23 (65.7)	3 (30.0)	20 (80.0)	0.008^**^
Existence of formal care plan	67 (91.8)	32 (91.4)	35 (92.1)	0.623
Formal care plan is adequately updated (for those who have a formal care plan)	4 (50.0)	2 (50.0)	2 (50.0)	0.757
Given information about their condition (if visited a consultant in the last 12 months)	23 (32.4)	9 (26.5)	14 (37.8)	0.307
Patient involved as much as they would like in decisions about their care	23 (47.9)	1 (5.9)	22 (71.0)	<0.001^***^
Patient wishes and preferences taken into account by health and social care professionals	18 (40.0)	0 (0.00)	18 (60.0)	<0.001^***^

*Bolded values indicate statistical significance at the level of p < 0.05*.

*Indicate values that remained significant after a false discovery rate correction, with ^*^Indicating significance at the p < 0.050.

** *Indicating significance at the p < 0.010 level*.

*** *Indicating significance at the p < 0.001 level*.

*MS, Multiple Sclerosis; FND, Functional Neurological Disorder*.

*Values written in parenthesis are percentages*.

### Relationships With Care Professionals

The results of the relationships with care professionals domain are summarised in Table 3. A significantly higher proportion of FND respondents than MS respondents felt their needs were not understood by hospital consultants *(p* < *0.010)*, other healthcare professionals within the hospital *(p* < *0.001)*, and their GP *(p* < *0.010)*. They also reported significantly more that they felt they were not treated with respect and dignity by hospital consultants *(p* < *0.050)*, other healthcare professionals within the hospital *(p* < *0.050)*, and their GP *(p* = *0.001)*. This contrasts to no respondents with MS reporting feeling they were not treated with dignity and respect by any healthcare professional (apart from the hospital consultant). There were no significant differences between the patient groups in their relationships with healthcare professionals outside the hospital and social services.

**Table 3:** Patients reporting a problem regarding their relationships with health and social care professionals.

	**Patients reporting needs not met**			
	**Total**	**MS**	**FND**	
	***N (%)***	***n (%)***	***n (%)***	***p***
**Perception of whether their needs are understood by healthcare professionals**				
Consultants in the hospital	12 (20.7)	1 (3.7)	11 (35.5)	0.003^**^
Other healthcare professionals in the hospital	13 (28.9)	0 (0.0)	13 (50.0)	<0.001^***^
GP	15 (23.1)	2 (6.9)	13 (36.1)	0.005^**^
Other healthcare professionals outside the hospital	10 (32.3)	2 (15.4)	8 (44.4)	0.092
Social services	8 (34.8)	1 (25.0)	7 (36.8)	0.565
**Treated with dignity and respect by healthcare professionals**				
Consultants in the hospital	10 (15.9)	1 (3.4)	9 (26.5)	0.013^*^
Other healthcare professionals in the hospital	7 (14.0)	0 (0.0)	7 (25.0)	0.012^*^
GP	11 (16.9)	0 (0.0)	11 (30.6)	0.001^**^
Other healthcare professionals outside the hospital	6 (24.0)	0 (0.0)	6 (40.0)	**0.028**
Social services	4 (19.0)	0 (0.0)	4 (25.0)	0.304

*Bolded values indicate statistical significance at the level of p < 0.05*.

* Indicate values that remained significant after a false discovery rate correction, with ^*^ indicating significance at the p < 0.050.

** *Indicating significance at the p < 0.010 level*.

** *Indicating significance at the p < 0.001 level*.

*MS, Multiple Sclerosis; FND, Functional Neurological Disorder*.

*Values written in parenthesis are percentages*.

### Community Care and Support

Problems reported by respondents involving aspects of their community care and support are summarised in Table 4. Significantly more respondents with MS reported problems in not receiving financial support for their neurological condition *(p* < *0.010)*. However, more FND respondents reported that their personal finances were affected by their neurological condition to a large extent *(p* < *0.001)*. A higher proportion of FND patients reported not being offered respite care, despite needing it *(p* < *0.010)*. There were no significant differences between patient groups in regards to accessing paid employment, occupational support, and guidance in staying in, returning to, or leaving employment. Likewise, there were no significant differences found in difficulties obtaining equipment, accessing accommodation modifications, difficulties in applying for financial support, accessing domestic support, and accessing private healthcare.

**Table 4:** Patients reporting a problem regarding aspects of community care and support.

	**Patients reporting needs not met**			
	**Total**	**MS**	**FND**	
	***N (%)***	***n (%)***	***n (%)***	***p***
Have not been in paid employment within the last 3 years	29 (42.0)	9 (27.3)	20 (55.6)	**0.017**
Assessment of how their neurological condition affects their work	15 (38.5)	7 (30.4)	8 (50.0)	0.217
Occupational therapist liaised with employer	10 (27.8)	7 (35.0)	3 (18.8)	0.242
Guidance to stay in employment	13 (32.5)	5 (20.8)	8 (50.0)	0.054
Guidance to leave employment	13 (33.3)	7 (30.4)	6 (37.5)	0.645
Guidance to return to employment	22 (31.0)	8 (22.2)	14 (40.0)	0.105
Experienced difficulty obtaining equipment for their neurological condition	23 (76.7)	8 (80.0)	15 (75.0)	0.571
Access to modifications to accommodation for their neurological condition	17 (34.7)	5 (22.7)	12 (44.4)	0.112
Receipt of financial support for modifications to accommodation	3 (15.0)	0 (0.0)	3 (15.8)	0.850
Receipt of financial support for their neurological condition	14 (21.9)	11 (36.7)	3 (8.8)	0.007^**^
Ease of applying for financial support	38 (86.4)	10 (76.9)	28 (90.3)	0.235
Personal finances affected by neurological condition	34 (48.6)	9 (25.7)	25 (71.4)	<0.001^***^
Offered help with housework	20 (29.0)	7 (20.6)	13 (37.1)	0.130
Offered help with personal care	5 (6.8)	1 (2.8)	4 (10.8)	0.187
Offered respite care	14 (19.2)	2 (5.6)	12 (32.4)	0.004^**^
Have used private healthcare for their neurological condition	11 (15.1)	5 (13.9)	6 (16.2)	0.781

*Bolded values indicate statistical significance at the level of p < 0.05*.

*Indicate values that remained significant after a false discovery rate correction, with ^*^indicating significance at the p < 0.050.

** *Indicating significance at the p < 0.010 level*.

*** *Indicating significance at the p < 0.001 level*.

*MS, Multiple Sclerosis; FND, Functional Neurological Disorder*.

*Values written in parenthesis are percentages*.

### Information

The majority of FND patients (81.1%) indicated that they would have liked to receive more information in relation to their neurological condition, which was not statistically significant from the 64.7% of MS patients who wanted more information. Amongst the FND patients who indicated they wanted more information, the most frequently indicated information desired was information about FND itself (53%), treatment for FND (50%), self-management strategies (40%), alternative therapies (35%), and medication (30%).

### EQ-5D-5L

FND patients reported significantly more problems compared to MS patients in regards to their mobility (*p* = 0.028), self-care (*p* = 0.007), usual activities (*p* = 0.003), and pain/discomfort (*p* < 0.001). There were no significant differences between FND and MS patients on problems with depression/anxiety. FND patients also had significantly lower health scores (Mdn = 47) than MS patients (Mdn = 70).

## Discussion

This study aimed to explore the problems that people with FND experience in their care by using the quality requirements outlined in the NSF and comparing them to MS patients. As predicted, the results indicated multiple significant differences in the experiences of care between people with FND and with MS, with people with FND experiencing an overall higher frequency of problems across multiple domains of care. Four main themes of problems that were more prevalent in FND respondents were: (1) delayed and poor communication in diagnosis and treatment; (2) negative relationships with healthcare professionals; (3) difficulties in accessing services and support; and (4) the burden of poor care experienced by FND patients. Together, these results indicate that the current care provided to FND patients within the NHS is falling significantly short of the standards of care expected for LTNCs, highlighting potential areas for improvement.

### Delayed and Poor Communication in Diagnosis and Treatment

People with FND identified marked problems in the time taken to be referred and diagnosed, as well as problems in the communication of treatment information. They thus experienced more delays in referral and diagnosis, reporting more frequently that they waited over 6 months for a specialist referral and over 12 months to receive a definite diagnosis. Additionally, they also identified poor communication in regards to the side effects of medications that they were prescribed for their condition. These delays and poor communication thus suggest that people with FND often wait longer to receive specialist care and, when they do access this care, they may be insufficiently informed of the expected side effects of the treatment. This may have a significant impact on both the outcome of their FND and their engagement with their treatment.

Prompt diagnosis and treatment is key in FND, as well as in LTNCs in general. Stone et al. (29) highlighted the role of diagnosis as a therapeutic tool in itself for FND, with symptoms often remitting or becoming less severe once a patient understands their cause and the potential for treatment. Consequently, delays in referral and thus delays in receiving a definite diagnosis means that people with FND may have to live with treatable disability for longer periods of time and risk the severity of their symptoms worsening. Multiple studies have shown that delayed diagnosis and a longer duration of symptoms are indicators of poor prognosis in FND and other functional disorders, whilst a shorter duration of symptoms, early diagnosis, and a high satisfaction with care predict more positive outcomes (10, 13, 30, 31). It is therefore clear that prompt diagnosis and treatment is key to improving outcomes for FND. Yet the FND respondents in this sample experienced significant delays, suggesting that there are issues within the referral and diagnosis pathway for FND which have the potential to negatively impact the long-term outcomes of their condition.

Additionally, the EQ-5D-5L demonstrates that these delays are not due to people with FND presenting with lower levels of disability, as they indicate that people with FND actually report significantly higher levels of disability across the majority of domains for health-related quality of life and have an overall worse health score. This corroborates previous findings that FND patients experience high levels of disability that equal or exceed that of other LTNCs (8, 9). Although these levels of disability are self-reported, it highlights that these patients are subjectively experiencing significant impairments within their daily lives, indicating a need for care, and support. Therefore, regardless of whether the severity of these disabilities are objectively different in the eyes of healthcare professionals, there should still be support offered to these patients to manage the impact that their disability has on their daily life.

Poor communication of medication side effects has been shown to be a problem within both physical and mental health contexts (32, 33), and has both ethical and practical implications for informed consent and the risk of adverse effects (34). More people with FND than with MS reported receiving inadequate information about the side effects of their prescribed medications, suggesting that these patients were potentially not able to make a fully informed decision about their-medical care and did not experience the same standard of communication from their healthcare providers that people with MS did. This is perhaps explained by the lack of evidence-based medication available across the spectrum of FNDs (35) and healthcare professionals' general lack of confidence and training in FND (36–38).

### Poor Relationships With Healthcare Professionals

People with FND also reported significantly more problems in their relationships with healthcare professionals. A substantial proportion of people with FND reported that they felt that their GP, consultant, and other healthcare professionals within the hospital neither understood their needs nor treated them with respect, something which only a few people with MS reported. This likely is linked to a significant lack of patient-centred care that these results also highlighted, as they also reported that they did not feel involved in their treatment decisions and felt that their wishes and preferences about managing their FND were not considered. Thus, a lack of patient-centred care for people with FND may have negative consequences on the relationships between these patients and healthcare professionals.

Issues with implementing patient-centred care, a vital component in improving patient outcomes (39, 40), may be in part explained by the stigma and negative perceptions attached to the FND diagnosis. For example, consultants have previously reported that they found people with FND more difficult to treat and support (41). Additionally, multiple studies have identified negative perceptions of FND in various healthcare professionals, including neurologists, psychiatrists, GPs, and nurses (18, 42–44). Lehn et al. (38) survey of healthcare practitioners linked these negative perceptions to a fundamental lack of knowledge or misunderstanding of what FND is. This is likely due to the precise aetiology of FND still remaining uncertain, although more recent research has begun to highlight the role of interacting psychological and physiological mechanisms (45, 46). Multiple studies have shown that people with FND report feeling stigmatised by healthcare professionals (47) and have had negative experiences during their care as a result, including feeling ignored, blamed, or humiliated in their interactions with professionals (21, 48). Patients in Lehn et al. (38) study attributed these negative experiences to healthcare professionals invalidating their experience and removing their autonomy over care decisions, which have also been echoed in the current results.

These results thereby suggest a need to develop more patient-centred care that removes stigmatising and negative experiences in the care of FND patients. Simple education for all healthcare professionals involved in the care of FND patients may be a beneficial and cost-effective step toward decreasing the stigma and negative perceptions attached to FND. For example, Lehn et al. (49) found that a brief educational course for healthcare professionals, created through multidisciplinary collaboration, was effective in improving the knowledge and confidence of professionals in assessing and supporting patients with FND, with this effect still present at 6 months follow up. Further, Rosendal et al. (50) developed an educational programme to educate GPs about the assessment and management of functional disorders in general and found that the programme decreased GP's negative attitudes toward functional patients and increased their satisfaction with interacting with these patients. Educating professionals may thus improve the relationships between patients and healthcare professionals.

### Difficulties Accessing Services and Support

Significant difficulties were experienced by people with FND in accessing services and support. They reported significant problems with the accessibility of healthcare professionals and specialist clinics, with a majority reporting that they had not been to a specialist clinic within the past year. They also highlighted issues in accessing respite care despite feeling they needed it. These difficulties may relate to the negative attitudes toward FND which may limit the responsiveness of healthcare professionals to the needs of these patients. For example, Ahern et al. (44) found that a substantial minority of neurology nurses with negative perceptions of people with FND symptoms also thought that they did not deserve the same level of care as patients with organic symptoms and were less willing to spend time caring for them. Although these were self-reported attitudes, there is an implication that these attitudes may actually translate into less responsive care for FND patients.

The difficulties in accessing services and healthcare professionals may also reflect problems in the structure and coordination of care services for FND and LTNCs in general, as a significant proportion of people with FND reported a lack of coordination between services in planning their care. Jackson et al. (51) found that people with LTNCs viewed coordination between services as vital in ensuring that there is a continuity of care within community settings, but that they often experienced limited access to these services. Since FND is often managed within community settings and requires multidisciplinary treatment (14), it is thus vital to have coordination between different services which should include, for many patients, a coordination between community neurotherapy and community mental health services.

### Burden of Illness

The consequences of the difficulties experienced by people with FND in these results is demonstrated through the burden of illness they also experience. The majority of people with FND in our sample felt that their personal finances were affected by their neurological condition to a large extent, despite them actually reporting significantly less problems in receiving financial support. The reason for this apparent paradox is unclear, although it is perhaps due to people with FND being unaware that they are able to access financial support, and/or that they often rely more on their own finances for their care due to a lack of confidence in public health and social care services. Sowińska and Czachowski (52), for example, found that people with functional symptoms in often have low expectations of their conditions being treated by healthcare professionals within public services, and instead sought treatment from private healthcare practitioners. As Stone (53) points out, some people with functional symptoms may disengage from healthcare services due to the stigma and lack of support they experience, suggesting that these patients may thus have to rely on informal methods of care, potentially having a negative impact on their personal finances to a larger extent than other LTNCs. Thus, people with FND may experience a greater burden (both financial and emotional) from their condition as a result of stigma and structural service issues.

The burden of care was also illustrated by the majority of people with FND (81.1%) desiring more information about their condition. Although this was not significantly different from MS patients, it suggests that most people with FND within our sample felt that they lacked information about their condition, with the most frequently desired categories of information being information about FND, treatment for FND, self-management strategies, alternative therapies, and medications available for FND. Since these patients do not feel adequately educated about these topics regarding FND by health and social care professionals, it is thus up to the patient to seek out information from other sources, increasing the burden on these patients and making them vulnerable to potential misinformation. This thereby emphasises the need for patient-centred care and educational resources for these patients.

## Limitations

There are important limitations to this study that may restrict the generalisability of the results. The relatively small sample size (*N* = 77) and that fact that the audit was only conducted at a single hospital means that replicating these findings in a larger sample across multiple neurology services would be important in validating the results of this current study. It would also be useful in identifying whether these problems are present across all neurology services or if specific characteristics of services, including management approach and level of specialism, may influence the frequency and type of problems reported. This would be important in elucidating the contextual factors that cause the problems reported in this study, thus providing more specific recommendations for service improvement. The patients in this study also only responded at one time point, and thus it is unclear how their experiences may have changed after contact with tertiary neurology services and over longer periods of time. This is particularly relevant as the patients presenting to the clinics in this study were referred primarily for further management planning of their condition. Thus, exploring the ways in which reported problems change after contact with neurology services may provide a more longitudinal view of FND patients' experiences throughout their contact with varying NHS services, from more general primary care services to more specialist neurology services. Further, more control groups are needed using a wider range of psychiatric and neurological conditions, as well as other types of functional disorders, to parse out the specific difficulties experienced by people with FND.

## Conclusions

These results suggest that negative attitudes and perceptions of FND from healthcare professionals, along with a general lack of structured care pathways for FND, may contribute to a greater burden and poorer experience of care for these patients. There are significant issues in the care of FND patients that require an integrated, systematic approach to address and mitigate these problems.

This may require the implementation of a stratified care model, as proposed by Healthcare Improvement Scotland (22), wherein there is a clear care pathway that directs health professionals and patients toward appropriate treatment depending upon need. Although specialist FND clinics remain rare, Aybek et al. (54) found them to have an important role in delivering accurate diagnoses and ensuring patients had access to appropriate care, and patients were found to be responsive to integrated neuropsychiatric management. Thus, the development of specialist multi-disciplinary FND clinics has the promise of improving both access to care and patient experiences. Gilmour et al. (14) noted that improving the knowledge and skills of community-based healthcare professionals and primary care workers is a vital step in developing effective, interdisciplinary stepped-care pathways for FND, which further highlights the role of education in improving care for FND patients. NHS England (55) has emphasised the importance of involving patients in the development of care pathways, suggesting there is also a need for both more research into FND patients' experiences of healthcare and the use of patient steering groups in order to deliver genuine patient-centred care.

The development of FND-specific care pathways that utilise interdisciplinary working, patient-centred care, and a stratified-care approach may therefore have significant potential in improving the experience of people with FND within the care services.

## Data Availability Statement

The raw data supporting the conclusions of this article will be made available by the authors, without undue reservation.

## Ethics Statement

Ethical review and approval was not required for the study on human participants in accordance with the local legislation and institutional requirements. Written informed consent for participation was not required for this study in accordance with the national legislation and the institutional requirements.

## Author Contributions

All authors listed have made a substantial, direct and intellectual contribution to the work, and approved it for publication.

## Conflict of Interest

The authors declare that the research was conducted in the absence of any commercial or financial relationships that could be construed as a potential conflict of interest.
